# Perinatal outcomes among Indian-born mothers in Australia

**DOI:** 10.1186/s12884-023-05897-8

**Published:** 2023-08-21

**Authors:** Kanmani Barthasarathy, Louisa Lam

**Affiliations:** 1 McCarthy Learning, Dandenong, VIC Australia; 2 School of Nursing, Midwifery and Paramedicine (VIC), Australian Catholic University, Sydney, VIC Australia; 3 Public Health and Preventive Medicine, Monash University, 553 St Kilda Road, Melbourne, VIC 3004 Australia

**Keywords:** Perinatal mortality, Perinatal outcomes, Low birth weight

## Abstract

**Objective:**

To examine the incidence of adverse perinatal outcomes and the risk of adverse perinatal outcomes for Indian-born mothers compared to other mothers living and giving birth in Australia.

**Design, setting and participants:**

This retrospective cohort study was designed to investigate all births in Australia in 2012 and those in the Monash Health Birthing Outcomes System (BOS) 2014 to Indian-born mothers in Australia. Data sets were analysed involving descriptive statistics using Statistical Package for Social Sciences (SPSS vs. 23).

**Results:**

Indian-born mothers in Australia are at increased risk of induced labour, emergency caesarean section, very preterm birth (20–27 weeks), babies with low to very low birth weight, and low Apgar score (0–2) at 5 min, gestational diabetes, hypothyroidism, iron deficiency anaemia and vitamin B12 deficiencies compared to other mothers giving birth in Australia. This is despite a range of protective factors (25–34 years, married, nonsmokers, and a BMI < 30) that would normally be expected to reduce the risk of adverse perinatal outcomes for mothers giving birth in a developed country.

**Conclusion:**

In the absence of many of the recognized maternal risk factors, Indian-born mothers continue to face increased risk of adverse perinatal outcomes, despite access to high quality maternity care in Australia. Recommendations arising from this study include the need for an intervention study to identify maternal risk factors for Indian-born mothers in mid to late pregnancy that contribute to the risk for very preterm birth and low birth weight.

## Statement of significance (SOS)

Australia boasts one of the safest maternity care systems in the world, with very low rates of maternal and perinatal mortality. It is, therefore, unexpected that immigrant Indian-born mothers continue to experience increased risk for adverse perinatal outcomes despite access to world class maternity care. No studies have examined perinatal outcomes for Indian-born mothers in Australia. The findings of this current study will measure the incidence of this increased risk compared to other mothers and identify the risk factors associated. This current study will contribute to existing knowledge on adverse perinatal outcomes, informing maternity care service providers of the specific risks faced by Indian-born mothers.

## Problem or issue

While numerous studies have reported on maternal risk for adverse perinatal outcomes, very few have considered maternal ethnicity and country of birth as a risk in itself. The purpose of this study is to identify the incidence of adverse perinatal outcomes for Indian-born mothers compared to other mothers living and giving birth in Australia. The study will investigate whether internationally agreed pregnancy risk factors for adverse perinatal outcomes used in mainstream maternity care can be usefully applied to predict risk for Indian-born mothers.

## What is already known

Developed countries like Australia, with increased migration are challenged by greater risk of adverse perinatal outcomes among Indian-born mothers compared to locally born mothers. Little is known about the impact that maternal country of birth and ethnicity has on perinatal outcomes.

## What this paper adds

This paper fins that adverse perinatal outcomes were more frequent among Indian-born mothers compared to other mothers living and giving birth in Australia in the absence of recognised maternal risk factors.

## The implications

Further intervention study is required to identify maternal risk factors in mid to late pregnancy that contribute to the risk of low birth weight and emergency caesarean section among Indian-born mothers.

## Introduction

Maternal country of birth has been identified as a potential risk factor for adverse perinatal outcomes including low birth weight and premature birth [1]. Studies report an association between the cultural impact of migration and maternal stress during pregnancy, an association also contributing to adverse perinatal outcomes [2]. Australia provides universal access to internationally recognised, quality maternity services as measured by national maternal and perinatal mortality rates which rank Australia among the best in the world [3]. However, it appears that access to quality maternity services does not protect Indian-born mothers from an increased incidence of adverse perinatal outcomes when giving birth in Australia [4].

Studies related to perinatal outcomes in Australia that include immigrant women have an emphasis on specific sub-immigrant populations using smaller sample sizes [5]. Based on ethnicity, international studies report significant disparities in perinatal outcomes [6] when associated with immigrant status [7]. Hannah Grace Dahlen, in a New South Wales study, stated that Indian-born mothers had a much higher rate of having private health insurance than the general population [2]. Her study demonstrated the link between low risk primiparous women giving birth in private hospitals and higher rates of surgical birth and obstetric intervention rates [2].

Australia benefits from the best global standards health indicators, reflecting the achievement of the lowest infant mortality rate in the world at 3.1 per 1,000 live births [8]. However, there exist disparities within Australian populations. In many health indicators, Indigenous Australians have disparities compared to non-Indigenous Australians [9]. Surprisingly, despite the stellar performance of the Australian maternity care system, Indian-born mothers are not benefiting as expected. Factors associated with this increased risk for Indian-born mothers must be identified to inform future maternity care advances in Australia. The issue of maternal ethnicity as a risk for adverse birth outcomes is explored. The Australian maternity service’s ‘one size that fits all’ model of care provision will be questioned in relation to the care provided to low risk, healthy, young, Indian-born mothers. The aim of this retrospective cohort study was designed to identify the risk of adverse perinatal outcomes of Indian-born mothers compared to other mothers.

## Materials and methods

### Study design and participants

The retrospective cohort study linked routinely collected deidentified data for all mothers giving birth to babies in Australia from January to December 2012, and at Monash Health in the State of Victoria in 2014.

### Data sources and linkage

The National Perinatal Data Set (NPD) from the Australian Institute of Health and Welfare (AIHW) National Perinatal and Epidemiology Statistics Unit (NPESU) which undertakes national reporting of reproductive and perinatal health information and statistics in Australia was used for this study. The data collected is based on births reported to the perinatal data collection in each state and territory in Australia [10] and obtained from the Maternity Information Matrix (MIM), a web-based summary of data items that includes 45 vastly different data collections [11]. The MIM is a snapshot of maternal information in Australia. As the national data was obtained in aggregated form and did not provide information relating to maternal medical conditions, past history, birth defects, neonatal morbidity, or obstetric complications, this current study utilised non-aggregated data solely from Monash Health (BOS) for 2014.

The second data set came from Monash Health. The Monash Health Birthing Outcomes System (BOS) data set for the year 2014 was obtained as non-aggregated data from Monash Health, which is a part of the Victorian State Perinatal Collection (VPDC). Data regarding mothers’ socio-demographic details, maternal country of birth, health conditions before and during pregnancy, complications during labour and birth, and perinatal outcomes, such as maternal and newborn health status after birth are collected [12].

In 2012, of “the 312,153 national births, of which 2,255 were stillbirths, the average maternal age was 30 years, with the youngest 15 years and the eldest 56 years”. Of women who gave birth in Australia in 2012, 31.2% were born in countries other than Australia. Regarding parity, 42.4% of mothers had their first baby and 33.2% had their second baby. In 2012, 62.7% of women attended at least one antenatal visit in the first trimester (before 14 weeks gestation), and 14.9% did not begin antenatal care until after 20 weeks gestation. Hospitals are the place of birth for almost all mothers (96.9%). Of all the women who gave birth, 19.4% had a caesarean section without labour and 12.9% had a caesarean section with labour. Of women who gave birth in hospitals in 2012, the proportion in private hospitals was 29.0%. Also, 6.2% of live born babies were of low birthweight (less than 2,500 g). In addition, 1.7% of live born babies had a low Apgar score (between 0 and 6) at 5 min.

In 2014, the number of mothers who delivered at Monash Health was 3,172. Of these births, 3 were stillbirths. The average maternal age was 30 years, with the youngest at 15 years and the eldest at 56 years. Of women who gave birth at Monash Health in 2014, 61.8% were born in countries other than Australia. Regarding parity, 32.5% of mothers had their first baby and 13.1% had their second baby. In 2014, 19.3% of babies were admitted to special care nurseries, and 2.0% were admitted to neonatal intensive care units. Of all the women who gave birth,15.2% had an emergency caesarean section and 14.4% had an elective caesarean section. Of women who gave birth at Monash Health in 2014, the proportion of mothers who smoked during pregnancy was 10.1%. Also, 3.4% of live born babies were of low birthweight (less than 2,500 g). In addition, 1.6% of live born babies had a low Apgar score (between 0 and 6) at 5 min.

**Fig. 1:**
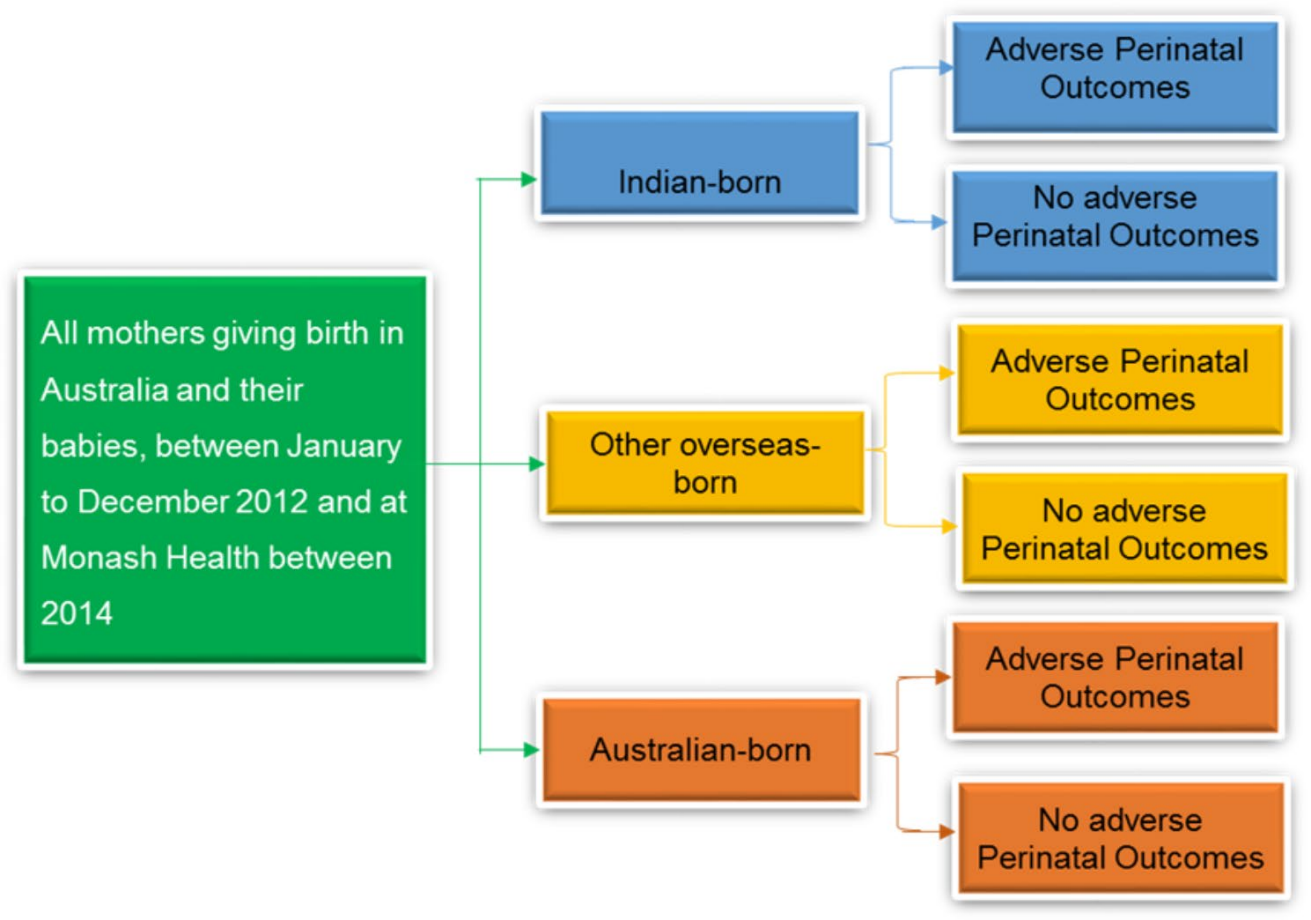
Proposed Conceptual Approach to Analysis

Two data sets were used, but there was no information about the population who were of Indian descent but born in Australia. Consequently, it was not possible to identify outcomes for Indian mothers born in India compared to women of Indian descent born in Australia or other overseas countries in both data sets. Thus, the differences between mothers who were **Indian-born** living in Australia were examined and compared to mothers who were born in other overseas countries, namely **other overseas-born mothers**, and mothers born in Australia namely, **Australian-born mothers**, both latter groups also potentially containing mothers with an Indian background. Acknowledging this ethnic limitation, these three groups were used to effect comparisons. The following Fig. 1 illustrates the proposed conceptual approach to the analysis.

### Data analysis

The statistical analyses for this study included the use of descriptive and inferential statistics. Descriptive statistics were used to determine the demographic and perinatal outcomes of the study populations (Indian-born, Australian-born, and other overseas-born mothers). They yielded frequencies, numbers, and percentages for each variable, using graphs as applicable. Inferential statistics, namely both parametric and non-parametric analyses, were employed to examine the association among variables. For categorical variables, cross-tabulations with a Pearson’s Chi-Square test were calculated to assess the association between each variable and a mother’s country of birth. Given the number of analyses performed, a P < 0.001 level of significance was used to minimise the possibility of Type 1 error. Effect size was calculated for all variables and analyses performed. Kruskal Wallis tests were calculated to determine any statistically significant differences, and the individual effect size was also calculated and compared for Indian-born mothers, Australian-born mothers, and other overseas-born mothers. The initial overview of the study results includes demographic characteristics and resource logistics from the overall data sets. Table 1 summarises the findings from the analysis of the NPD and Table 2 summarises the findings from the analysis of the Monash Health (BOS) Hospital data.

**Table 1:** Maternal Characteristics and Outcomes Among Indian-born, Australian-born and Other Overseas-born Mothers (2012) NPD (National Perinatal Data)

Variables	Pearson Chi-Square	df	p	Effect size V
Mothers				
Maternal age	7,336.43	10	< 0.001	0.10
SEIFA IRSD	365.81	2	< 0.001	0.04
Maternal parity	2,677.70	8	< 0.001	0.06
Plurality	64.32	2	< 0.001	0.01
Maternal marital status	5,293.02	4	< 0.001	0.09
Maternal smoking status	7,815.41	2	< 0.001	0.16
Maternal BMI (kg/m^2^)	2,386.14	6	< 0.001	0.08
Pregnancy at first antenatal visit in weeks	1,533.46	2	< 0.001	0.05
Number of antenatal visits	413.35	10	< 0.001	0.03
Intended place of birth	135.24	6	< 0.001	0.02
Actual place of birth	204.30	6	< 0.001	0.02
Hospital sector	2,047.30	2	< 0.001	0.08
Method of birth	1,194.14	6	< 0.001	0.04
Onset of labour	675.81	4	< 0.001	0.03
Perinatal Outcomes - Babies				
Presentation	18.82	6	< 0.001	0.01
Birth status	3.90	2	0.143	0.00
Gestational age (in weeks)	517.81	2	< 0.001	0.02
Birth weight (grams)	2,545.29	2	< 0.001	0.07
Apgar score (at 5 min)	7.74	2	0.021	0.02

**Table 2:** Maternal Characteristics and Outcomes Among Indian-born, Australian-born and Other Overseas-born Mothers (2014) MHD (Monash Health (BOS) Data)

Variables	Pearson Chi-Square	df	p	Effect size V
Mothers				
Maternal age	131.62	10	< 0.001	0.14
Maternal parity	93.93	8	< 0.001	0.12
Plurality	1.00	2	0.606	0.02
Maternal marital status	306.35	4	< 0.001	0.22
Substance abuse	318.14	14	< 0.001	0.22
Maternal medical conditions	318.14	14	< 0.001	0.22
Past history	423.39	44	< 0.001	0.21
Method of birth	47.77	8	< 0.001	0.09
Onset of labour	29.89	4	< 0.001	0.007
Perinatal Outcomes - Babies				
Presentation	13.13	6	< 0.001	0.05
Birth status	1.24	2	0.538	0.02
Gestational age (in weeks)	2.98	2	0.225	0.03
Birth weight (grams)	63.27	2	< 0.001	0.11
Apgar score (at 5 min)	0.27	2	0.872	0.01
Admission to special care nurseries or neonatal intensive care units	17.42	6	0.008	0.05
Neonatal morbidity	100.76	48	< 0.001	0.13
Birth defect	33.49	22	0.055	0.07
Obstetric complications	160.55	48	< 0.001	0.16

### Ethical approval

The Monash University Human Research Ethics Committee (MUHREC) and the Monash Health Human Research Ethics Committee (MHHREC) approved this study. No informed consent was required from any individuals as the study used existing de-identified datasets obtained with approval from the Australian Institute of Health and Welfare (AIHW) National Perinatal and Epidemiology Statistics Unit and from Monash Health for the Birthing Outcomes System (BOS) data set.

## Results

**Fig. 2:**
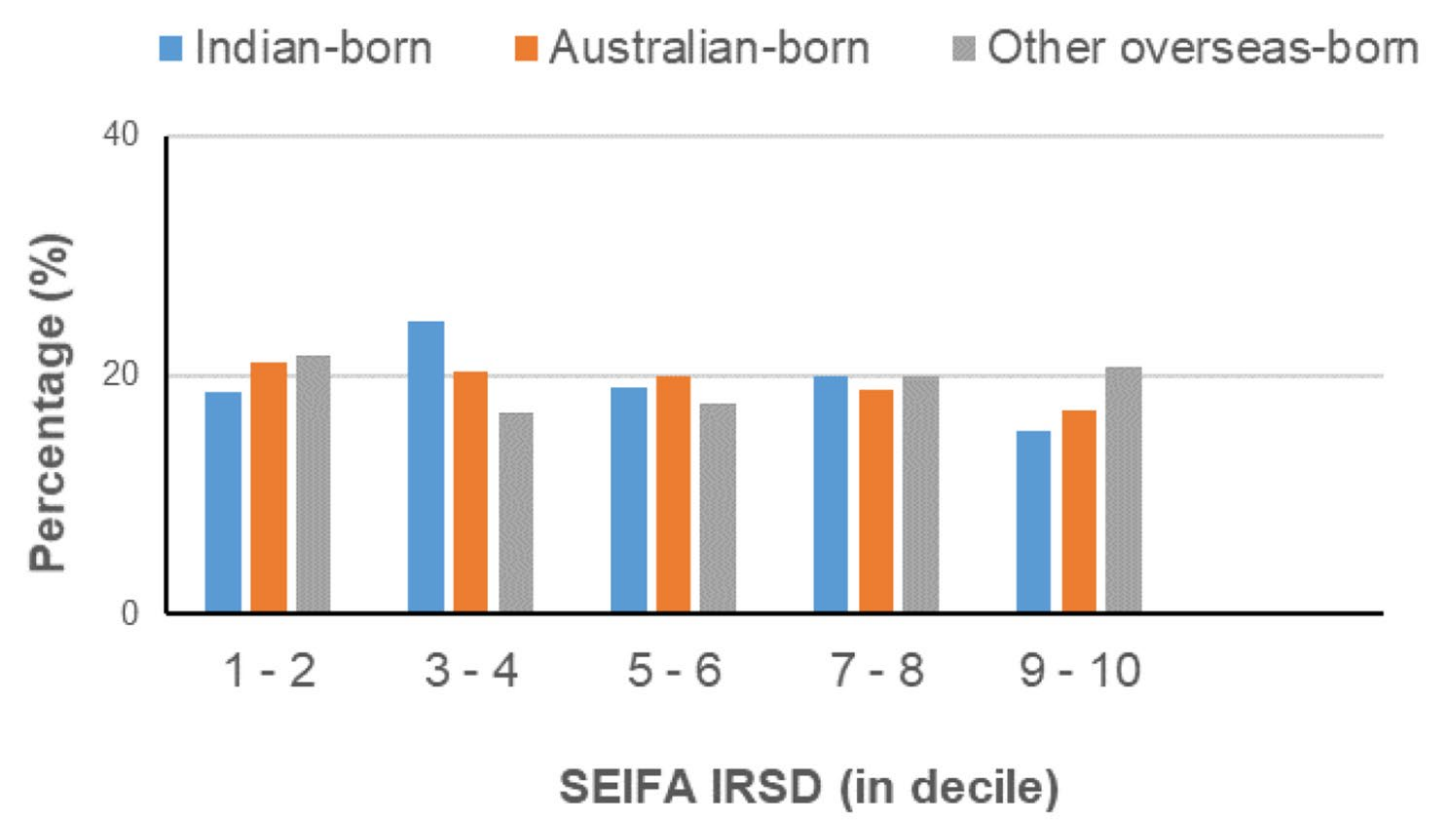
SEIFA IRSD (in decile) and Maternal Country of Birth (NPD)

The largest proportion of mothers aged 25–29 and 30–34 giving birth in 2012 were Indian-born mothers (i.e., 45.38% & 37.39%), as compared to Australian-born mothers (27.62% & 30.90%), and to other overseas-born mothers (26.04% & 36.02%) (refer Table 3). The percentage who were married was larger for Indian-born mothers (97.9%) than for Australian-born mothers (81.91%) and other overseas-born mothers (90.07%) (refer Table 4) and the proportion who reside in lower socio-economic areas with a Socio-Economic Indexes For Areas Index of Relative Socio-Economic Disadvantage (SEIFA IRSD) index between 3 and 4 decile (24.48%), when compared to (20.39%) of Australian-born mothers, and (16.95%) of other overseas-born mothers (Fig. 2). Indian-born mothers were least likely to smoke during pregnancy (0.28%), compared to Australian-born mothers at (16%) and other overseas-born mothers at (5.25%). A BMI of 30 + was smallest among Indian-born mothers at (10.48%), this being the case for (13.31%) of other overseas-born mothers, and (21.28%) of Australian-born mothers. This is the case across both the National Perinatal Data set 2012 and the Monash Health (BOS) Data set 2014. The proportion of singleton births was largest among Indian-born mothers, limiting the influence of multiple births as a potential contributor to low birth weight in this group of mothers. Despite a slightly delayed presentation for the first antenatal visit, Indian-born mothers were more likely to attend all recommended antenatal visits [10–13] when compared to other mothers.

**Table 3:** Maternal Age (in years) and Mothers’ Country of Birth (NPD VS MHD)

Maternal age (in years)	Indian-born				Australian-born				Other overseas born			
	n		%		n		%		n		%	
	NPD	MHD	NPD	MHD	NPD	MHD	NPD	MHD	NPD	MHD	NPD	MHD
< 20	12	0	0.12	0.0	9,904	57	4.61	4.7	1,219	20	1.40	1.3
0–24	783	33	7.60	8.4	33,059	196	15.38	16.2	8,422	176	9.69	11.2
**25–29**	**4,673**	**149**	**45.38**	**37.9**	**59,394**	**361**	**27.62**	**29.8**	**22,638**	**440**	**26.04**	**28.1**
**30–34**	**3,850**	**174**	**37.39**	**44.3**	**66,448**	**335**	**30.90**	**27.7**	**31,320**	**587**	**36.02**	**37.4**
35–39	860	30	8.35	7.6	37,295	204	17.35	16.8	18,652	268	21.45	17.1
>=40	118	7	1.15	1.8	8,856	58	4.12	4.8	4,681	77	5.38	4.9
Not stated	1	-	0.01	-	53	-	0.02	-	13	-	0.01	-
Total	10,297	393	100	100	215,009	1211	100	100	86,945	1,568	100	100

Note. NPD = National Perinatal Data; MHD = Monash Health (BOS) Data

**Table 4:** Marital Status and Maternal Country of Birth (NPD VS MHD)

Marital status	Indian-born				Australian-born				Other overseas-born			
	n		%		n		%		n		%	
	NPD	MHD	NPD	MHD	NPD	MHD	NPD	MHD	NPD	MHD	NPD	MHD
**Married (including de facto)**	**10,082**	**386**	**97.91**	**98.2**	**176,118**	**796**	**81.91**	**65.7**	**78,312**	**1375**	**90.07**	**87.7**
Widowed/divorced/ separated	22	0	0.21	0.0	2,449	17	1.14	1.4	1,053	18	1.21	1.1
Never married	95	7	0.92	1.8	33,050	398	15.37	32.9	6,175	175	7.10	11.2
Not stated	98	-	0.95	-	3,392	-	1.58	-	1,405	-	1.62	-
Total	10,297	393	100	100	215,009	1211	100	100	86,945	1568	100	100

Note. NPD = National Perinatal Data; MHD = Monash Health (BOS) Data

Indian-born mothers reported a very low rate of substance abuse during pregnancy, as compared to Australian-born mothers. Data related to maternal medical conditions was routinely not collected across the Australian states and territories for the national data set and, therefore this data was not included in the 2012 data set. Table 2 shows the details of chi-square, significance p value and effect size for each variable. These variables were included in the 2014 hospital BOS data set. Among the medical conditions recorded, the largest proportion affecting Indian-born mothers involved anaemia, diabetes mellitus, Vitamin D deficiency, Vitamin B12 deficiency, polycystic ovary syndrome, and hypothyroidism when compared to other mothers who gave birth in 2014. The percentage of babies admitted to special care nurseries requiring observation, blood glucose monitoring, and experiencing neonatal jaundice was largest among Indian-born mothers when compared to other mothers. Musculoskeletal birth defects were also higher among babies of Indian-born mothers. It is interesting to note that the proportions of mothers suffering from anxiety, depression, asthma, cervical dysplasia and increased BMI > 30 were largest among Australian-born mothers.

Indian-born mothers were most likely to give birth in a public hospital, and most likely to have a previous caesarean section when compared to other mothers. Indian-born mothers were most likely to have an induction of labour and a caesarean section birth, an increased risk of Apgar score (0–2) @ 5 min, prematurity (gestational age 20–27 weeks), and low to very low birth weight < 1000 g-1.16% & 2000–2499 g than other mothers. Indian-born mothers also have a slightly larger risk of stillbirths compared to other mothers. Tables 1 and 2 show the details of chi-square, significance p value and effect size for each variable in both data sets. Most of the variables were statistically significant. However, some non-significant variables expressed a slight statistical difference.

The largest proportion of blood glucose monitoring was among neonates to Indian-born mothers at 15.0%, with 9.3% for the neonates of Australian-born mothers. The proportion for other overseas-born mothers’ neonates was 10.1%. The rates of neonates with jaundice, undergoing observation, and other issues, were higher among neonates of Indian-born mothers who gave birth during 2014 (i.e., jaundice-0.7%; observation only-11.7%; others-9.4%), when compared to Australian-born mothers’ neonates (i.e., jaundice-8.3%; observation only-10.5%; others-7.3%) and to other overseas-born mothers’ neonates (i.e. jaundice – 9.5%; observation only–9.0%; others–6.8%).

## Discussion

Specific research into Indian-born mothers’ perinatal outcomes has been limited in the Australian context. In general, most published perinatal research examines general comparisons among immigrant mothers, refugee mothers, and Indigenous Australians [13]. Immigrant Indian-born mothers do not share many of the socioeconomic disadvantages experienced by other immigrant groups, particularly refugees. Indian immigrants migrate to Australia as part of the skilled migrant scheme which involves employment in low to middle income work [14]. The literature review demonstrated a lack of population-based studies specifically investigating Indian-born mothers and their adverse perinatal outcomes in Australia. Of the few publications reporting adverse perinatal outcomes for Indian-born mothers in host countries, most investigate mortality risk among babies and obstetric intervention rates [4]. Also, studies indicate that most migrant mothers have increased risks for gestational diabetes mellitus compared to mother residents in receiving countries [15]. A recent study in Australia has confirmed that South Asian Indian-born mothers have a greater risk for late pregnancy stillbirth, low birth weight, and induced labour compared to Australian-born and New Zealand-born mothers [16]. Findings from the current study add to and nuance these earlier results.

In the current study, a majority of Indian-born mothers gave birth between 24 and 34 years of age, considered to be the optimal age for childbirth [17]. Indian-born mothers had fewer teenage pregnancies and fewer mothers aged over 35 years compared to Australian-born and other overseas-born mothers. Studies in the US and Norway reveal that the birth rates of women 35–39 and 40–45 years have doubled in the last 30 years [18]. Therefore, other risk factors must be investigated to explain why they are at increased risk for adverse perinatal outcomes in Australia [16].

Australia provides free maternity care for all mothers who give birth in public hospitals [19]. It has been suggested that Australian maternity care reflects a ‘one size fits all’ model that may not cater for culturally diverse beliefs pertaining to pregnancy and childbirth [20]. Immigrant mothers residing in developed countries often experience worse pregnancy outcomes requiring targeted attention to improve the antenatal care they receive [21]. Consequently, other risk factors must be investigated to explain why they are increased risk for adverse perinatal outcomes in Australia. Indian-born mothers having a parity of one or two is considered to be a protective factor against risk for adverse perinatal outcomes [22]. Raatikainen et al. reported that marital status is a protective factor for adverse perinatal outcomes [23]. The majority of Indian-born mothers giving birth in Australia are socioeconomically advantaged through marriage (refer Table 4). In the absence of increased risk associated with socioeconomic disadvantage, other risk factors need to be identified that explain why Indian-born mothers are at increased risk for adverse perinatal outcomes.

In Australia, the prevalence of smoking is highest for Indigenous mothers than for others [24]. Previous research has also shown that smokeless tobacco impacts pregnancy, with decreasing gestational age at birth and low birth weight independent of gestational age [25]. Indian-born mothers are non-smokers and yet have low birth weight babies. In a recent study comparing weight gain during pregnancy (using Institute of Medicine Guidelines) among Asian Indians across different body mass index (BMI) categories, mothers who gained less weight than recommended had a low risk for caesarean section an increased (but statistically insignificant) risk for low birth weight and preterm birth [26]. The majority of Indian-born mothers are of healthy weight throughout pregnancy but remain at increased risk for adverse birth outcomes in Australia.

Indian-born mothers have a higher rate of induced labour and caesarean section birth or instrumental birth than in other mothers giving birth in Australia [16]. Induction of labour is currently practised for medical indications, such as prolonged pregnancy and prolonged rupture of membranes [27], and is performed in approximately 20% of low risk pregnancies [28]. Increased numbers of Indian-born mothers had a forceps or vacuum extraction birth [12], which was double the number of Australian-born and other overseas-born mothers. Caesarean section birth was more frequent among Indian-born mothers than Australian-born and other overseas-born mothers. Rates of emergency caesarean section birth are increased in certain migrant groups e.g. Indian, African and Latin American [29]. These increased rates are unexplained, and do not reflect national trends [12].

Potential factors affecting Indian born mothers?

**Fig. 3:**
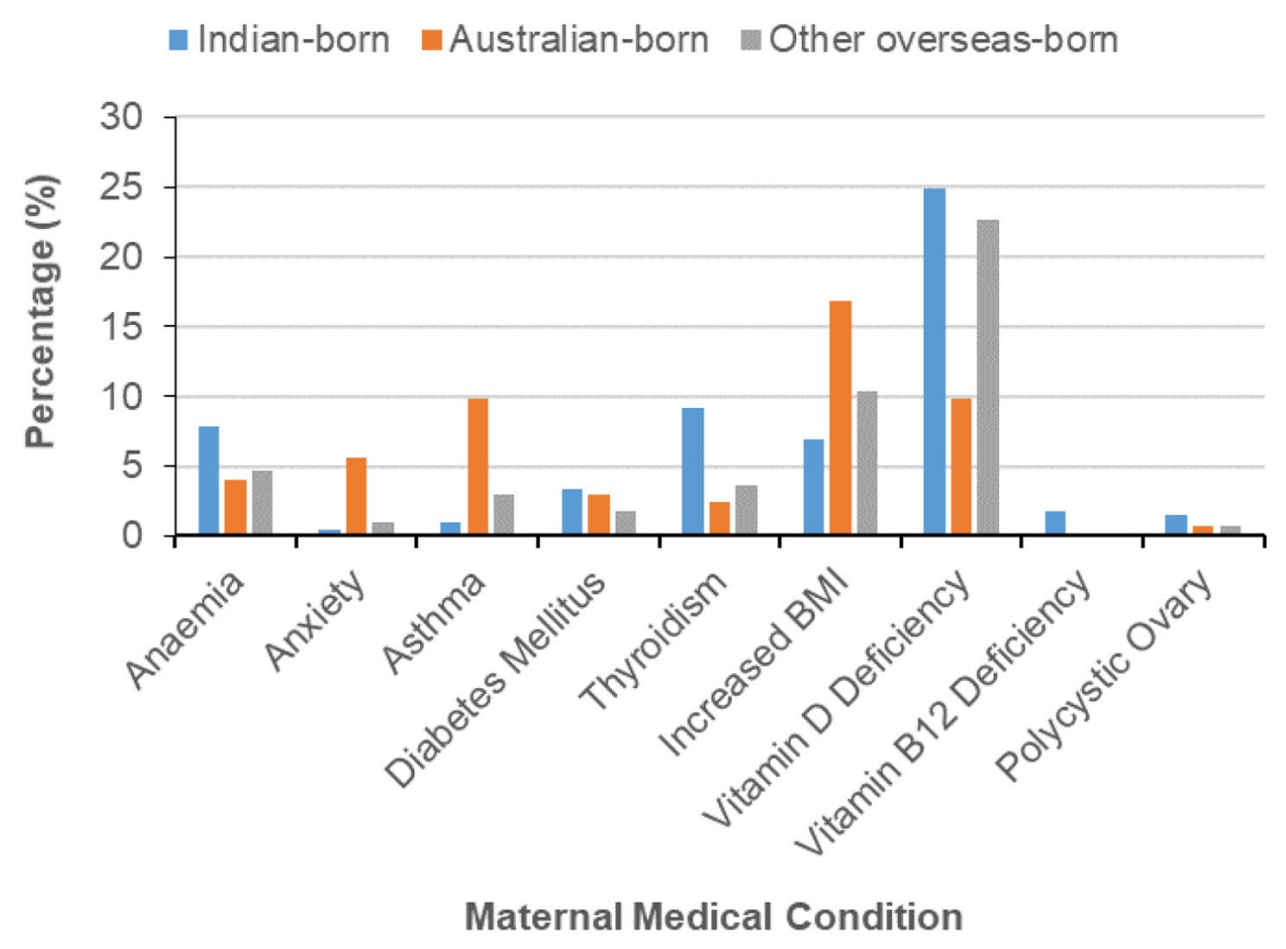
Maternal Medical Condition and Country of Birth (MHD)

- This current study reveals that when compared to Australian-born and other overseas-born mothers a largest proportion of Indian-born mothers were found to have iron deficiency anaemia (Table 5) (Fig. 3). These findings are supported by previous research in Australia by Fernandez et al. (2015), who reported that Indian-born mothers are more prone to iron deficiency anaemia [30].

**Table 5:** Maternal Medical Conditions and Country of Birth (MHD)

Variables	India-born		Australia-born		Other overseas-born	
	n	%	n	%	n	%
***Maternal medical conditions***						
Anaemia	31	7.9	49	4.0	74	4.7
Anxiety	2	0.5	68	5.6	16	1.0
Asthma	4	1.0	119	9.8	46	2.9
Auto-Immune Disease	0	0.0	9	0.7	4	0.3
Bi–cornuted Uterus	2	0.5	4	0.3	4	0.3
Cancer of Cervix	0	0.0	2	0.2	0	0.0
Cardiac Condition	2	0.5	26	2.1	16	1.0
Chronic Bowel Disease	1	0.3	11	0.9	1	0.1
Chronic Renal Disease	0	0.0	3	0.2	4	0.3
Depression	3	0.8	45	3.7	24	1.5
Diabetes Mellitus	13	3.3	36	3.0	26	1.7
Endometriosis	1	0.3	8	0.7	4	0.3
Epilepsy	3	0.8	9	0.7	5	0.3
Fibroids	2	0.5	10	0.8	17	1.1
Genital herpes	0	0.0	7	0.6	4	0.3
Thyroidism	36	9.2	29	2.4	57	3.6
Increased BMI	27	6.9	204	16.8	162	10.3
Others	19	4.8	89	7.3	95	6.1
Vitamin D deficiency	98	24.9	119	9.8	355	22.6
Vitamin B12 deficiency	7	1.8	2	0.2	1	0.1
Urinary tract infection	1	0.3	2	0.2	2	0.1
Polycystic ovary	6	1.5	9	0.7	11	0.7
Not stated	135	34.4	351	29.0	640	40.8
Total	393	100	1,211	100	1,568	100

Note. MHD = Monash Health (BOS) Data

Gestational diabetes mellitus is a more common maternal medical condition among India-born mothers in Australia [31]. South Asian mothers are more prone to pre-existing diabetes and gestational diabetes mellitus than Australia-born and New Zealand-born mothers [16]. These findings are consistent with the international trends for increased rates of gestational diabetes mellitus among mothers migrating from low to high income countries including mothers of Indian and Arab ethnicity [32].

- Vitamin D deficiency: Vitamin D is important for placental function and necessary for optimal fetal growth and development [33]. Indian-born mothers have increased rates of vitamin D deficiency (Table 5) (Fig. 3).
- Vitamin B & B12 deficiency: Vitamin B12 is essential for normal growth and development of the fetus during pregnancy [34]. South Asian mothers have an insufficient dietary B12 intake [35] associated with vegetarianism or low meat eating practices [35]. This is supported by a New Zealand study that found that B12 deficiency is common in South Asian childbearing age mothers in Auckland [36].
- Thyroidism: Indian-born mothers are at increased risk for developing hypothyroidism resulting in maternal thyroid dysfunction and adverse birth outcomes [37]. Adverse birth outcomes can be prevented by early detection and management of hypothyroidism [38]. *Polycystic ovary*: Polycystic ovarian syndrome affects Indian-born mothers at a greater rate than other women, the cause for which is unexplained [39].

There is emerging evidence that implicates maternal ethnicity as a factor associated with premature placental aging [40]. Indian-born mothers also have a significantly smaller placental surface area, weight and volume than other mothers of Asian descent [41]. It has been suggested that the optimal birth time for South Asian mothers is 38–39 weeks, before complications associated with placental aging begin to impact the wellbeing of the fetus [42]. Similarly 38–39 week birth is recommended for African-Caribbean mothers to avoid late pregnancy risk for fetal compromise [43].

This current study was unable to identify the proportion of healthy small babies whose weight was in the higher ranges of the low birth weight < 2,499 g. The inclusion of small healthy babies may be responsible for the very high incidence of low birth weight reported for Indian-born mothers. Dahlen et al. measured Indian-born mothers’ risk for low birth weight to be twice that of Australian-born mothers [4]. Alarmingly, India accounts for 40% of low-birth-weight cases in the developing world [44]. Indian-born mothers have a similar increased risk for low birth weight and babies born small for gestational age when living in the United State Of America and Canada compared to Caucasians and African Americans [45]. Placental monitoring including measurement of placental diameter and placental volume, is recommended in late pregnancy in addition to other routine fetal surveillance for women where suboptimal fetal growth is suspected [46].

The current study makes some unique contributions. The findings reveal that Indian-born mothers possess many protective factors that are expected to minimise adverse perinatal outcomes, such as being married, singleton plurality, access to antenatal care involving qualified maternity care professionals, normal BMI, non-smoking and lack of substance abuse during pregnancy, and giving birth in a public hospital fully equipped to manage complications. Despite all these protective factors, Indian-born mothers giving birth in Australia still face an increased risk for adverse perinatal outcomes. Indian-born mothers are at greatest risk for preterm birth, low birth weight and very low Apgar @ 5 min.

Findings from the current study confirm that Indian-born mothers living and giving birth in Australia are at increased risk of adverse perinatal birth outcomes. Placental monitoring from mid to late pregnancy is suggested [16]. Vitamin D is important for placental function and necessary for optimal fetal growth and development [33]. Indian-born mothers have increased rates of vitamin D deficiency. Vitamin D deficiency is routinely managed using oral vitamin D supplements for cases where serum 25-hydroxyvitamin D3 concentrations are below 20–40 nmol/L [47]. Pre-pregnancy intervention is required to address this avoidable vitamin deficiency by increasing foods rich in vitamin D in the regular diet.

Low dietary vitamin B-12 intake in the presence of high total folate intake is a marker of adverse birth outcomes for Indian mothers [48]. South Asian mothers have an insufficient dietary B12 intake [35] associated with vegetarianism or low meat eating practices [35], combined with higher prices for food containing B12 [49]. Approximately 40–75% of Indian-born mothers of childbearing age are affected [34, 50]. This is supported by a New Zealand study that found that B12 deficiency is common in South Asian childbearing age mothers in Auckland [36]. The presence of dietary deficiency diseases in Indian–born mothers living in Australia is of concern and requires urgent community attention. Indian-born mothers are at increased risk for avoidable dietary deficiencies resulting in iron deficiency anaemia, vitamin D and vitamin B & B12 deficiency and hypothyroidism due to low iodine intake (Table 5) (Fig. 3). Eradicating these avoidable conditions must be a priority in reducing risk for adverse perinatal outcomes. Scant Australian studies have reported on the relationship between dietary supplements and birthweight among Indian-born mothers. Those that do, report that Indian-born mothers are more prone to iron deficiency anaemia [30]. The nutritional status of Indian-born mothers after migration to Australia is unclear. It is, therefore, necessary to assess the nutritional level of Indian-born mother before, during, and after pregnancy. A further recommendation related to the need for antenatal care provided to Indian-born mothers be tailored, to manage specific risks (maternal medical conditions) contributing to increased risk for adverse perinatal outcomes.

### Limitations

This study analysed routinely collected birth data sets, these data sets were not collected specifically for research questions. Another limitation of this study is that it is restricted to Indian-born mothers who gave birth in Australia in 2012 and at Monash Health hospitals during 2014. Further, the data sets do not report some pre-existing maternal medical, obstetric, and gynaecologic factors necessary to address the research questions. Also, the 2012 National Perinatal Data and 2014 Monash Health (BOS) hospital Data sets were collected for another purpose, unrelated to this study, and were collected in different years. Moreover, only first generation Indian-born mothers could be identified in both data sets.

## Conclusion

This research is the first of its kind in Australia that investigates Indian-born mothers’ risk for adverse perinatal outcomes as a discrete group. The study makes a significant and timely contribution to the specific risks faced by Indian-born mothers, risks that are not routinely considered. This current research confirms that Indian-born mothers are at an increased risk for adverse perinatal outcomes compared with other mothers having babies in Australia. The Australian maternity service is renowned for the universal provision of high quality maternity care by qualified health professionals [3], but we need to be mindful that this Indian population needs to be considered in identifying effective clinical models with additional care. However, it is a strong recommendation of this study that maternal ethnicity be recognised as a risk for adverse perinatal outcomes, and that Indian-born mothers be recognised as an at-risk group for the purposes of antenatal care birth.

## Data Availability

The datasets generated and/or analysed during the current study are not publicly available due to the data were used under license for the current study but are available from the corresponding author on reasonable request” and with permission of [Faculty of Medicine, Nursing and Health Sciences School of Nursing & Midwifery].
